# To compare anti-albumin urea effects of valsartan alone with combination of valsartan and amlodipine in patients of chronic kidney disease

**DOI:** 10.12669/pjms.323.9541

**Published:** 2016

**Authors:** Ameen, Muhammad Ali Kashif, Sumreen

**Affiliations:** 1 Dr. Ameen, FCPS. Department of Medicine, Combine Military Hospital (CMH), Bahawalpur, Pakistan; 2 Dr. Muhammad Ali Kashif, FCPS. Department of Medicine, Combine Military Hospital (CMH), Bahawalpur, Pakistan; 3 Dr. Sumreen, M.Phil. Department of Pathology, SMBBMC, Department of Medicine, Combine Military Hospital (CMH), Bahawalpur, Pakistan

**Keywords:** Albuminuria, Amlodipine, Spot urinary albumin: creatinine, Valsartan

## Abstract

**Objective:**

To compare anti-albumin urea effects of Valsartan alone with combination of Valsartan and Amlodipine in patients of chronic kidney disease.

**Methods:**

This randomized clinical trial was conducted at the Department of Medicine, Combined Military Hospital Bahawalpur, from April 2014 to 30 September 2014. 140 patients of chronic kidney disease with baseline blood pressure more than 140/90mm Hg having raised urinary albumin: creatinine ratio (UACR). UACR more than 3.5 mg/mmol was considered abnormal. Group-A was treated with Valsartan 80mg daily and Group-B was treated with valsartan 80 and amlodipine 10mg once a day. We did not change the dose of drugs and check spot UACR at base line and after six months with therapy and compare improvement in UACR between Group-A and B. Data was analyzed by statistical software packages (SPSS 16.0).

**Results:**

In both the groups, BP was significantly lower than the respective value. Mean decrease in spot UACR in Group-A was 3.18±2.64 mg/mmol and UACR in Group-B mean decrease in UACR was 13.01±20.11 mg/mmol. P value was< 0.05.

**Conclusion:**

The combination therapy of valsartan with amlodipine significantly lowers the albuminuria in chronic Kidney disease and reduce the progression of disease as compared to Valsartan alone therapy.

## INTRODUCTION

Chronic kidney disease (CKD) is a heterogeneous group of disorders characterized by alterations in kidney structure and function, which manifests as Glomerular filtration rate (GFR) <60ml/min/1.73m^2^ for more than three months with or without kidney damage.^1^ Prevalence of CKD in Pakistan is 12.5%.^1^ CKD is mainly caused by hypertension and diabetes, other important causes are glomerulonephritis, polycystic kidney disease and lupus nephritis.^2^ Good blood pressure (BP) control prevent progression of CKD.^2^ Proteinuria is one of the clinical parameters to diagnose renal damage particularly in glomerular hypertension.^3^ The suppression of proteinuria is a major goal in the treatment of hypertensive patients with chronic kidney disease, which prevent renal parenchymal injury.^3^ Many trails have been done to prevent progression of kidney disease by reducing proteinuria, which is a basic factor in reducing morbidity and mortality in CKD.^4^

Many drugs have been tried to reduce progression of diabetic nephropathy and hypertension, but Angiotensin receptor blockers (ARBs) have shown best result. Therefore ARBs are first line drugs in diabetic nephropathy.^5^

The renin-angiotensin system (RAS) regulates blood pressure and fluid balance in the body, 3 when blood volume or sodium levels in the body are low, RAS produces enzyme Angiotensin II. Angiotensin II stimulates the release of the hormone aldosterone in the adrenal glands, which causes the renal tubules to retain sodium, water and excrete potassium.^3^ Angiotensin II causes vasoconstriction thus increased BP and aldosterone raise blood volume, sodium levels in the blood.^3^ Blockade of RAS with angiotensin receptor blockers (ARBs) is currently the most effective strategy for renal-protection. In the end stage CKD, accumulation of toxins, fluid, and electrolytes normally excreted by the kidneys results in the uremic syndrome. This syndrome could lead to death unless the toxins are removed by renal replacement therapy, using dialysis or kidney transplantation. Progression from mild to moderate CKD to end stage chronic kidney disease (ECKD) can be delayed by controlling hypertension and proteinuria. However, monotherapy is not sufficient to control BP, particularly in patients with CKD, highlighting the need for combination drug therapy with calcium channel blockers (CCBs).^6^ Amlodipine is L-type CCB which is more effective to reduce proteinuria than other CCB.^7^ ARBs and amlodipine reduces BP and intra glomerular pressure which decrease protein filtration at glomerulus so this combination decrease proteinuria in CKD patients.^3^ A study conducted by Krzesinski JM et al. showed Valsartan, Amlodipine combination is effective to reduce proteinuria in CKD.^8^

ARBs and ACE inhibiters are beneficial as first line antihypertensive agents for hypertension in patients with proteinuria but, they are unable to maintain the blood pressure at a level below 130/80 mmHg, as is required in patients with chronic kidney disease (CKD),^7^ for such BP maintenance, second anti-hypertensive agent is required. ACE inhibiters are associated with dry cough and so we choses ARBs for our study. This study was planned to compare anti-albumin urea effects of Valsartan alone with combination of Valsartan and Amlodipine in patients of chronic kidney disease with the help of spot UACR.

## METHODS

This randomized clinical trial was conducted at the department of Medicine, Combined Military Hospital Bahawalpur, from April 2014 to 30 September 2014. Approval from hospital’s Ethical committee was obtained. Written consent was taken from all participants. Diagnosed patients of Chronic kidney disease with persistant uncontrolled hypertension, BP more than 140/90 mmHg and Albuminuria (urinary albumin: creatinine ratio >3.5 mg/mmol) were included in the study. Patients with history of heart failure, angina, myocardial infarction in last six months, patients with immunosuppressive therapy, endocrine hypertension and uncontrolled diabetes with ketoacidosis were excluded. A total of 140 patients who fulfilled inclusion criteria were enrolled in the study by using non-probability purposive sampling. Patients’ age range was 20 to 70 years. Patients were assigned into two groups. Group-A treated with Valsartan 80 mg once a day (ARBs) and Group-B treated with Valsartan 80 mg and Amlodipine 10 mg once a day(ARBs+CCB). Groups were allotted by random allocation software version 1.0.0. The doses of the ARBs and CCBs were not be altered during the study period. The target BP level was < 130/80 mmHg. First morning void urine sample was collected for spot UACR. Urine albumin was analyzed using Albumin Tinaquant Roche/Hitachi reagent kit, based on immunoturbidimeteric technology. Urine creatinine was measured by the Jaffé assay using Beckman UniCel® DxC 600 Synchron® Albumin creatinine ratio (ACR) was calculated by dividing the urinary albumin concentration with urinary creatinine concentration. The spot urinary albumin: creatinine ratio was measured before and after six month of treatment in both groups and then we compared net improvement in proteinuria between Group-A and B. A detailed structured Performa was used to collect the data.

Data was analyzed by statistical software packages (SPSS 16.0). Qualitative variables like sex present as frequency and percentage. Quantitative variables age, spot UACR was analyzed using mean and standard deviation. Independent (unpaired) t-test was used to compare mean change in UACR post treatment between two groups. P-value ≤ 0.05 was considered as significant. Independent (paired) t-test was used to compare pre and post treatment UACR with in a group.

## RESULTS

There were 78 (55.71%) male and 62 (44.2%) female patients. Age distribution is given in Table-I.

**Table-I:** Age distribution (n=140).

*Age (in years)*	*Group-A (n=70)*		*Group-B (n=70)*	

	*No. of patients %*		*No. of patients %*	
20-45	07	10.0	18	25.7
46-70	63	90.0	52	74.3
Total	70	100	70	100
Mean±SD	56.33±8.203		53.09±9.821	

P-value-0.0360

The baseline mean UACR of Group-A was 28.29±6.75mg/mmol and of Group-B patients’ was 43.46±7.56 mg/mmol. Both groups were treated with selected drugs for 6 months period. After treatment the mean UACR in Group-A was 25.11±8.71 mg/mmol and in Group-B was 30.45±9.45 mg/mmol detail is given Table-II.

**Table-II:** Comparison of reduction in UACR pre and post treatment.

	*UACR 1*	*UACR 2*	*P value*
Group-A (N=70)	28.29±6.75	25.11±8.71	0.005
Group-B (N=70)	43.46±7.56	30.45±9.45	0.001

Paired t–test with in a group demonstrated there were significant reduction in albuminuria in both groups but patients in Group-B showed a marked reduction in albuminuria, with a mean difference of 13.01±2.11 mg/mmol, between pre and post treatment, which is statistically significant, p-value 0.001. Whereas in Group-A, albuminuria reduction was 3.18±2.04 mg/mmol, with significance of, p- value 0.005. Unpaired t-test was used to compare mean reduction in UACR after the treatment between groups given in Table-III.

**Table-III:** Comparison of mean reduction in UACR between Group-A and B after treatment.

*Group-A (N=70)*	*Group-B (N=70)*	*P-value*
3.18±2.04 mg/mmol	13.01±2.11 mg/mmol	0.001

## DISCUSSION

Hypertension and diabetic nephropathy are leading causes of CKD in Pakistan and associated with the morbidity and mortality related to CKD.^9^,^10^ Prevalence of Hypertension is 22% in Pakistan.^10^ The presence of conventional risk factors along with poor control of blood pressure among the patients with CKD highlight the need to integrate CKD prevention and management in the primary care hospital in Pakistan.^11^ Our study showed that ARBs + CCBs more efficiently reduce albuminuria in CKD patients than ARB monotherapy.

Our findings are consistent with study conducted by Abe M et al 2010 in Japan, which demonstrated that addition of Cilnidipine (CCBs) with ARBs result in good blood pressure control, reduction in albuminuria, and decrease the progression of CKD.^3^

Recently a study carried out in China by Dayi HU, proved a single pill combination of ARB and CCB was more effective than other drugs in reducing blood pressure as well as proteinuria in CKD.^12^ The BP-lowering efficacy of Valsartan/ Amlodipine in a single pill combination was independent of age and comorbidities. BP control of less than 140/90 mmHg was achieved in 76.8% of the patients and reduction in protean urea was independent form sex and age. This study is in agreement with our study which proved that combination of Valsartan and Amlodipine reduced protein urea in all patients irrespective of age and sex.

Trials on hypertensive patients have proven that more than 80% of these patients need more than one drug to control BP and those with renal failure, have benefited with combination therapy due to improvement in GFR. Our results are also comparable with this study.^8^

In the study performed by Yilmaz MI in Turkey 2010, diabetic patients with Stage-1 CKD treated with either amlodipine (10mg/day) or valsartan (160mg/day), or their combination. The result showed improvement in proteinuria with combination therapy was remarkable than single therapy alone.^13^ In another study conducted by Kaneshiro Y and fellow, the add-on effect of amlodipine to patients taking valsartan resulted in marked improvement in UACR as well as on reduction of BP and pulse wave velocity.^14^

Takashi Ono in 2013 conducted a study over the efficacy of different ARBs in reducing albuminuria which showed Olmesartan (ARB) significantly decreased daily urinary protein compared with the other ARBs.^15^

On the basis of above it is recommended that, CKD patients with hypertension should be regularly monitored with spot UACR and early prophylaxis should be offered with valsartan and amlodipine combination. It will help to prevent morbidity and mortality due to CKD and prevent progression to end stage CKD which required renal replacement therapy.

There are few limitations of our study. It is single center experience with limited sample size so result could not be applied to whole population; therefore further work is required with multi-centric approach.

## CONCLUSION

Valsartan and Amlodipine combination is associated with significant reduction in albuminuria as compare to Valsartan alone. Combination therapy is more efficient to reduced proteinuria.
